# 

**Published:** 1896-10-10

**Authors:** 


**Notices of Births, Deaths, and Marriages** are inserted in this column at a charge of 2s. 6d. for an announcement not exceeding four lines.

### NOTICE TO CORRESPONDENTS.

All MS., Letters, Books for Review, and other matters intended for the Editor should be addressed The Editor, The Lodge, Porchester Square, London, W.

The Editor cannot undertake to return rejected MS., even when accompanied by stamped directed envelope.

**Notice to Advertisers and Subscribers.**

All Advertisements, Orders for Copies of The Hospital, and Business Communications should be addressed to **THE MANAGER, (Not to The Editor),** The Hospital, 28 & 29, Southampton Street, Strand, London, W.C.

The Cost of Subscription, post free, is as follows:—Per week, 2½d.; per quarter, 2s. 8d.; per half-year, 5s. 3d.; per annum, 10s. 6d. Foreign:—Per annum, 15s. 2d.; payable, in all cases, in advance.

**IMPORTANT NOTICE.**

On and after October 1st, 1896, the EDITORIAL and PUBLISHING OFFICES of "THE HOSPITAL" will be TRANSFERRED to their new premises, 28 & 29, SOUTHAMPTON STREET, STRAND, LONDON, W.C.

**NOTICE.—Vol. XX. of "The Hospital" (April to September, 1896), in handsome cloth binding, will be on sale at the Office of this Journal, early in October, price 7s. 6d. Cases for binding the Numbers contained in this Volume, 1s. 6d. Index to Vol. XX., 2½d., post free.**

**The Monthly Part for October is now ready, Price 6d., by post 8d.**

### SCRAPS AND GLEANINGS.

It is believed that Swansea Hospital will benefit to the extent of £340 by the recent carnival.

The Guardians of the Paddington Workhouse are about to erect new lunacy wards at a cost of £3,700.

Ninety-five cases of accident were treated at the Blackpool Hospital during the year ended August, 1896.

About £2,000 is required to provide suitable laundry accommodation for the Dunoon Convalescent Homes.

A plan is on foot to erect an infectious hospital for the three townships of Horsforth, Rawdon, and Guiseley.

There will be 40 instead of 16 beds when the new wing is completed at the Monkwearmouth and Southwick Hospital.

It is stated that a Polish engineer has invented a portable crematory for incinerating the bodies of those who die in battle.

The annual concert promoted by the East Ward Committee of the Leeds Workpeople's Hospital Fund realised £15 5s. 4d.

The total amount realised by the Woking Hospital Saturday collection this year was £206, which is an increase on last year's takings.

The number of patients admitted to the Brighton, Hove, and Sussex Throat and Ear Hospital during the month of August was 120.

The total number of cases treated during the last year at the Monkwearmouth and Southwick Hospital was 4,181, against 4,150 last year.

The total cost of the new infirmary at Bridgnorth has been £6,099 against which £5,727 has been received, leaving a deficit of about £371.

The amount realised at the annual service at St. Peter's Church, Mansfield, on behalf of the Mansfield Woodhouse District Hospital was £75 10s.

A grand performance of "Elijah" by the Llanelly Harmonic Society is being arranged in aid of the funds of the Llanelly Hospital, which is £500 in debt.

About £650 is required to defray the cost of the repairs and ventilation of the West Norfolk and Lynn Hospital. Of this sum £440 has already been promised.

The cost of the new buildings recently opened in connexion with the Glasgow Samaritan Hospital has been £10,500, and, thanks to its good friends, it has been possible to open it free from debt.

The total number of patients who received medical and surgical assistance at the Stockton and Thornaby Hospital during the past year has been 2,476, which is the largest number since the present building was opened.

The committee of the Amalgamated Friendly Societies has handed to the treasurer of the Dover Hospital the sum of £280 towards the funds of that institution, being the result of their Hospital Saturday and other collections.

The number of persons received during the past year at the West of Scotland Seaside Convalescent Homes, Dunoon, was 4,056. Recently the homes have been greatly extended, and now 250 people can be accommodated at one time.

An international exhibition of the Grocery, Provision, Oil and Italian Warehouse and Allied Trades was held from October 2nd to October 9th. This was the first annual exhibition here of the kind, and it proved extremely interesting.

The total amount collected at various places of worship in the West Bromwich district for the past year in connection with the West Bromwich Hospital Sunday Committee showed a falling off of about £70, compared with the previous year.

At Elmira, New York State, there is a reformatory for converting into useful citizens criminals whose wickedness is the result of hereditary weakness or the outgrowth of a poisoned environment. The results are stated to be highly satisfactory.

The managers of the Johannesburg Hospital, South Africa, have just provided that institution with a complete set of Merryweather hand fire engines, which will be kept ready for use at all times, and the nurses and officials are to be drilled in their use.

The Aston District Council propose to purchase a site at Witton on which to erect a new small-pox hospital, the purchase to be conditional to the Local Government Board granting a loan for securing the land and the subsequent erection of buildings thereon.

The third annual collection in connection with Glasgow Hospital Sunday Fund takes place on November 29th. Already 240 churches and 109 Sabbath schools have intimated to the committee their intention of joining in the effort on behalf of the three great infirmaries.

It is stated that the Board of Management of the Hereford General Infirmary have decided to erect two isolation wards for males and females. It is considered that the outbreak of small-pox in 1888 might possibly have been prevented had there been means for complete isolation at the infirmary.

In New York there is an ordinance, which, however, is not enforced, providing that no handbill or advertisement giving notice of any person having skill in the treatment of any illness, or offering for sale any medicine, shall be permitted to be posted in any street of the city, under a penalty of 25 do's.

The amount received from the working classes in and around Belfast on behalf of the Belfast Royal Hospital during the past year showed a decrease of £83 on the previous year. This decrease is accounted for by the loss sustained by the long dispute in the engineering and shipbuilding trade, and other causes.

One thousand four hundred and twenty-eight children entered this year for the annual sports promoted amongst the West Ham school children for the purpose of securing funds to assist the children's ward in the West Ham Hospital. This may give some idea of the self-imposed labour entailed upon the teachers who form the Sports Committee.

The Ham Yard Soup Kitchen and Hospice, Ham Yard, Great Windmill Street, W., has, during the past year, provided 232,271 meals, 74,498 being penny dinners to sandwich men and others, the remainder being free by register, &c.; 1,020 families were supplied with a substantial Christmas dinner, and 191 tons of coal were distributed in half-hundred weights.

### Books Received.

J. and A. Churchill.

"Hints on Elementary Physiology." By Florence A. Haig Brown.
"A Manual for Hospital Nurses." By E. J. Domville.
"Observations on Congenital Dislocation of the Hip." By Bernard E. Brodhurst, F.R.C.S.

Oliver and Boyd.

"Royal Infirmary Cliniques." By Alexander James, M.D.

**Periodicals and Pamphlets.**—Charity Organisation Review, Therapist, Scalpel, American Journal of the Medical Sciences, Guy's Hospital Gazette, Pediatrics, Medical Bulletin, Woman's Signal, Public Health, Clinical Journal, London, Tri-State Medical Journal, Homœopathic Review, Westminster Review, Inter-Colonial Medical Journal of Australasia, Windsor Magazine, Indian Medico-Chirurgical Review, Medical Press, Literary Digest, United Temperance Gazette, Photogram, Nursing Notes, New York Medical Journal, Columbus Medical Journal, Leeds Hospital Magazine, Practitioner, Cornhill Magazine, Medical Pioneer, Bulletin of the Johns Hopkins Hospital, Medical Press, Medical Week, Charlotte Medical Journal, American Medico-Surgical Bulletin, New Saturday, Dietetic and Hygienic Gazette, Leisure Hour, Boy's Own Paper, Girl's Own Paper, Sunday at Home.

### The Student of Medicine.

### APPOINTMENTS.

W. F. Adams, M.R.C.S.Eng., L.R.C.P.Lond., appointed Junior Assistant Medical Officer at the Cornwall County Asylum at Bodmin. T. W. Arnison, M.B., Ch.B.Vict., appointed Junior House-Surgeon to the Salford Royal Hospital. G. Ashton, M.B., Ch.B.Vict., M.R.C.S., L.R.C.P., appointed House Surgeon to the Manchester Southern and Maternity Hospital. R. D. Dalal, L.R.C.P.Lond., M.R.C.S.Eng., L.M. and S., Bombay, appointed Resident Medical Officer of the Finsbury Dispensary. H. N. Edwards, M.R.C.S., L.R.C.P., appointed Clinical Assistant to the Chelsea Hospital for Women. T. Farthing, M.B., C.M.Edin., appointed Deputy Medical Superintendent to the Dartford Joint Hospital Committee. J. H. Glover, M.B., C.M.Edin., appointed Medical Officer for the Bottesford District of the Belvoir (Leicester) Union. J. P. Hall, M.B., Ch.B.Vict., M.R.C.S., L.R.C.P., appointed House Surgeon to the Salford Royal Hospital. G. H. Jenkins, L.R.C.P.E., L.R.C.S.E., L.F.P.S.G., appointed Medical Officer of Health to the Usk Urban District Council, Medical Officer of Health to the Pontypool Rural District Council, and Medical Officer and Public Vaccinator to the Usk District, Ponytpool Union. G. B. Mason, M.R.C.S., L.R.C.P.Lond., appointed Second Assistant Medical Officer to the St. Marylebone Infirmary. H. H. Phillips, M.R.C.S., L.R.C.P., appointed Clinical Assistant to the Chelsea Hospital for Women. W. R. Rice, M.D., appointed Lecturer on Human Physiology at the Coventry Technical Institute. J. B. Sutton, L.R.C.P.Lond., L.S.A., appointed Medical Officer for the Hedgerley District of the Eton Union. W. E. Thomas, L.R.C.P., L.R.C.S.Edin., appointed Medical Officer for the Pentre District of the Pontypridd Union. E. S. Warburton, M.R.C.S.Eng., L.S.A., appointed Medical Officer for the Treherbert District of the Pontypridd Union. R. A. Welsh, M.B., B.S.Durh., appointed Medical Officer for the Embleton District of the Alnwick Union.

### PASS LIST.

### Society of Apothecaries of London.

The following candidates passed in:

Surgery.—N. C. Collier, King's; A. P. B. Ellis, Glasgow; J. O. Garland, Guy's.

Medicine, Forensic Medicine, and Midwifery.—C. C. Brymer, Montreal; N. C. Collier, King's; H. Fulton, Guy's; H. A. J. Gidney, Calcutta; J. A. Williams, McGill.

Medicine and Forensic Medicine.—H. L. Billett, Royal Free; A. E. Shaw, Cambridge and St. Thomas's.

Medicine and Midwifery.—G. H. Smith, St. Bartholomew's.

Medicine.—W. Allen, Birmingham; W. A. Davidson, Birmingham and London; A. H. Grace, Bristol.

The diploma of the Society was granted to the following candidates: Messrs. Collier, Davidson, Ellis, Garland, Gidney, Smith, and Miss Billett.

### University College, London.

Medical Entrance Scholarships have been awarded to Mr. L. A. E. de Zilwa (£137 11s.), Mr. C. S. Parker (£57 15s.), and Mr. R. E. Lloyd (£57 15s.).

### Charing Cross Hospital Medical School.

Livingstone Scholarships (100 guineas) awarded to Mr. C. Jerome Mercier; Huxley Scholarship (55 guineas) awarded to Mr. F. B. Pinniger; Epsom Scholarship (110 guineas) awarded to Mr. L. C. Badcock; University Scholarships (60 guineas each) awarded to Mr. H. S. Clogg and Mr. R. J. Willson; Entrance Scholarships are also awarded to Mr. W. B. Blandy (60 guineas) and Mr. Charles H. Fennell (40 guineas).

### Guy's Hospital Medical School.

The examiners for the Open Entrance Scholarships in Science and Arts have made the following awards: In Science—Myers Coplans, Simon Langton School, Canterbury, Scholarship £150; John Ford Northcott, St. John's College, Cambridge, Scholarship £60; W. Blandy, Nottingham High School; F. Shufflebotham, Trinity College, Cambridge; F. H. Tully, Private Study; H. C. Keates, Guy's Hospital; and C. May, King's College, London, Certificates. In Arts.—W. Tuohy, Bedford Modern School, Scholarship £100; A. H. E. Wall, Wellington College, New Zealand, Scholarship £50; A. J. Gwalkin, Scholarship £30 (Dental School); J. B. Stephens, Christ's Hospital; A. H. Lewis, St. Olave's Grammer School; and L. G. Nash, Felsted School, Certificates.

